# Explorative team learning as a catalyst for innovation and performance: Evidence from Vietnam’s aviation sector

**DOI:** 10.1371/journal.pone.0360402

**Published:** 2026-10-30

**Authors:** Hanh Thi My Ly, Zuzana Tučková, Nhat Tan Pham, Thi Anh Van Nguyen, Hung Xuan Nguyen

**Affiliations:** 1 Hoa Sen University, Ho Chi Minh City, Vietnam; 2 Tomas Bata University in Zlín, Zlín City, Czech Republic; 3 School of Business, International University, Ho Chi Minh City, Vietnam; 4 Vietnam National University, Ho Chi Minh City, Vietnam; 5 Faculty of Economics, Ho Chi Minh City University of Technology and Engineering, Ho Chi Minh City, Vietnam; 6 Ho Chi Minh City University of Economics and Finance, Ho Chi Minh City, Vietnam; Institute of Management Sciences Peshawar, PAKISTAN

## Abstract

In today’s dynamic service sectors, particularly aviation and tourism, innovation is a vital driver of performance. While leadership is known to foster innovation, the mechanisms by which leadership influences innovation outcomes through team learning remain underexplored. This study examines the impact of innovation leadership on team performance through team explorative learning, employee innovation, and team innovation, and further examines the moderating role of customer relationship management (CRM) practices in these relationships. Drawing on Social Exchange Theory and Social Learning Theory, innovation is conceptualized as a multi-level outcome shaped by leadership and team dynamics. Using Structural Equation Modelling on survey data from 430 employees and leaders in Vietnam’s aviation and tourism sector, the findings show that innovation leadership enhances team explorative learning, which in turn promotes employee and team innovation. These innovation outcomes subsequently enhance team performance, confirming their mediating roles in the relationship between team explorative learning and performance. Moreover, customer relationship management (CRM) practices moderate these relationships by strengthening the effect of team innovation on performance and attenuating the direct relationship between team explorative learning and team performance. In contrast, CRM practices had a limited moderating effect on the relationship between employee innovation and team performance. This study advances understanding of the indirect pathways through which leadership drives innovation and highlights CRM practices as a boundary condition. Practical implications for managing innovation in complex service environments are offered.

## 1. Introduction

The aviation and tourism industries face unprecedented challenges, including intense competition in service quality and heightened vulnerability to external shocks such as economic crises and global pandemics [1]. Unlike many other sectors, services in aviation and tourism are highly perishable, customer experience-driven, and dependent on safety, reliability, and trust. In such a volatile environment, sustaining competitiveness requires organizations not only to maintain service quality and safety but also to innovate and adapt to evolving customer demands continuously [2]. Building such resilience requires effective training, communication, and learning environments that enhance organizational agility and knowledge integration [3,4]. Within this context, innovation leadership becomes indispensable, as leaders are expected to articulate a compelling, creative vision and inspire teams to explore, experiment, and embrace new ways of working [5].

A central mechanism through which leadership fosters innovation is team explorative learning. By encouraging knowledge sharing, experimentation, and openness to new approaches, explorative learning is associated with enhanced creativity, knowledge exchange, and adaptive problem-solving in uncertain environments [6]. When promoted, it allows members to exchange expertise, generate innovative ideas, and implement improvements at both the individual and collective levels [7,8]. Complementing this, customer relationship management (CRM) practices may serve as a contextual condition that enables organizations to leverage customer knowledge and feedback to support innovation and performance outcomes [9,10].

Despite its importance, a review of prior studies reveals several important gaps. First, research on innovation leadership has largely focused on the technology and education sectors [11,12], while its influence on team explorative learning in service-intensive, high-risk contexts such as aviation and tourism remains underexplored. Second, although prior work has demonstrated the impact of team learning on performance [13,14], the mechanisms through which team explorative learning translates into performance outcomes remain insufficiently specified, particularly regarding employee innovation and team innovation as distinct but related mediating pathways. Third, while some scholars have highlighted the relevance of CRM practices for innovation [9,15], empirical evidence positioning CRM practices as a moderating factor in the learning – innovation – performance relationship is still lacking, particularly in high-end service industries. Finally, despite the rapid recovery of Vietnam’s aviation sector following COVID-19, empirical studies are scarce on how innovation leadership, team learning, and innovation outcomes interact in emerging market contexts. Addressing these gaps is critical for aviation and tourism organizations, where competitiveness depends heavily on the ability to innovate while maintaining safety, service quality, and customer trust. Without such insights, firms risk falling behind in an industry where customer expectations evolve rapidly, and resilience against disruption is increasingly tied to innovation capabilities.

In response, this study develops and empirically tests a comprehensive model that examines how innovation leadership fosters team explorative learning and how this, in turn, influences team performance indirectly through employee and team innovation, with CRM practices as a boundary condition. The study contributes to theory by extending the application of innovation leadership research into the high-risk service domain, advancing understanding of the mediating and moderating mechanisms linking learning, innovation, and performance, and providing empirical evidence from a post-pandemic emerging market. The findings offer actionable insights for aviation and tourism organizations in Vietnam to enhance human resource development, strengthen service innovation, and improve operational quality in dynamic and uncertain environments.

## 2. Literature review

### 2.1. Theoretical background

#### Social exchange theory.

Social Exchange Theory (SET) explains organizational relationships in terms of reciprocal individual exchange [16,17]. Accordingly, positive actions from leaders or organizations, such as support, fairness, and recognition, create a sense of obligation and motivate employees to reciprocate with commitment, positive learning, and innovative behavior [18,19]. SET provides a strong theoretical basis for showing how innovation leadership encourages team explorative learning in high-contact service environments such as airlines and tourism, which require constant teamwork and creativity. From this perspective, innovation leadership can be understood as a source of social exchange that fosters team explorative learning, as supportive and inspiring leadership behaviours encourage knowledge sharing, collaboration, and engagement in learning processes [12,20]. These learning processes facilitate the application and refinement of knowledge, thereby supporting both employee innovation and team innovation, which in turn contribute to improved team performance. Additionally, CRM practices may reinforce social exchange relationships by enhancing interaction and visibility of knowledge, thereby shaping how learning and innovation processes translate into performance outcomes [15].

#### Social learning theory.

Social Learning Theory (SLT) suggests that people learn through observing the behaviour of others and direct or indirect social experiences [21,22]. Rather than relying solely on reinforcement mechanisms, SLT emphasizes that knowledge and skills can be acquired through observation, imitation, and social interaction within organizational contexts [23]. In organizations, this perspective explains how employees learn from leaders and colleagues, shaping behaviours related to collaboration, creativity, and innovation [24]. In high-contact service environments such as aviation and tourism, continuous interaction among team members facilitates observational and experiential learning, which supports the development of innovative behaviours and improved performance. Moreover, group interactions, knowledge sharing, and role rotation within teams further strengthen social learning, enabling employees to internalize effective practices and contribute to innovation. In such contexts, learning is often multidimensional, involving cognitive, behavioural, and emotional development, underscoring the importance of a supportive, trust-based learning environment [25,26].

### 2.2. Hypothesis development

#### Innovation leadership and team explorative learning.

Team learning is widely recognized as a key mechanism for organizational adaptation and success in dynamic environments [27]. It develops through processes of sharing, discussing, and reflecting on knowledge among members, which strengthens everyday awareness and coordination [28,29]. Within this domain, team explorative learning emphasizing the search for and discovery of new knowledge is particularly critical for driving creativity and innovation.

Drawing on Social Exchange Theory, innovation leadership can be viewed as a source of support and reciprocity that encourages employees to engage in learning-oriented behaviours. Leaders who promote knowledge sharing, build psychological safety, and support creative initiatives stimulate behaviours that advance team explorative learning [30,31]. Such leadership behaviours foster a climate of trust and knowledge exchange, which facilitates the development of learning oriented teams [32,33]. Moreover, Zaccaro et al. [34] demonstrated that innovative leaders who motivate team members, provide resources, and encourage exploration directly strengthen the process of team explorative learning. Taken together, these arguments suggest that innovation leadership plays a critical role in fostering team explorative learning. Therefore, the following hypothesis is proposed:

*H1: Innovation leadership has a positive impact on team explorative learning.*

#### Team explorative learning, team innovation, and employee innovation.

Team explorative learning refers to the process through which members proactively seek, experiment with, and develop new knowledge and skills in response to environmental change and emerging challenges [35]. This process is particularly critical in heterogeneous teams, where differences in expertise and experience provide diverse resources and perspectives, thereby facilitating more creative and innovative solutions [36,37]. From a Social Learning Theory perspective, explorative learning enables individuals to observe, experiment with, and internalize new ideas, thereby facilitating the development of innovative behaviours. Prior research shows that explorative learning enables teams to move beyond established routines and expand their search for knowledge, thereby supporting the generation and implementation of novel ideas [38,39]. These processes strengthen team-level innovation by enabling teams to transform newly acquired knowledge into collective outcomes such as new products, services, or processes.

Moreover, a learning-oriented climate fosters collaboration, psychological safety, and adaptability, all of which are vital for sustaining creativity in uncertain and dynamic environments [40,41]. These dynamics collectively support team-level innovation by equipping teams to design new products, processes, or services that meet evolving market demands. Explorative learning also plays a key role at the individual level by fostering employees’ innovative behaviour. When team members experiment, share, and reflect on new ideas, they are more likely to exhibit creative initiative in their daily work [42,43]. An explorative learning environment cultivates open-mindedness, stimulates self-reflection, and promotes individual willingness to innovate [44]. Furthermore, when employees feel empowered and supported in testing ideas, they are more likely to recognize opportunities for improvement and transform them into concrete innovations [45,46]. Building on this theoretical and empirical foundation, we propose:

*H2: Team explorative learning has a positive effect on team innovation.*

*H3: Team explorative learning has a positive impact on employee innovation.*

#### Team and employee innovation and team performance.

While team explorative learning equips members with new knowledge and skills, such learning does not necessarily translate into performance outcomes unless it is effectively applied in practice. Ideas must be transformed into concrete outcomes such as products, processes, or services to generate value. In this context, team innovation can be understood as a mechanism that links learning processes to performance outcomes by converting creative ideas into implementable results [47, 48]. Studies emphasize that explorative learning becomes meaningful only when it produces implementable innovations [38,39]. By generating and applying such innovations, teams maximize learning benefits and enhance overall performance [49].

Successful innovation further requires aligning internal capabilities with external demands [50], while fostering originality, unconventional thinking, and contributions that enrich organizational practice. From a Social Learning Theory perspective, team innovation reflects the collective application of learned knowledge, where team members transform shared experiences into coordinated innovative actions. Nevertheless, stimulating innovation within teams remains a leadership challenge, as balanced participation and knowledge sharing are essential for creative problem-solving and decision-making [51]. Frequent team interactions also enhance motivation and collective commitment, strengthening the likelihood that learning translates into performance outcomes [52]. These insights suggest that team innovation mediates the relationship between explorative learning and team performance. Taken together, these arguments suggest that team innovation serves as a mediating mechanism through which team explorative learning influences team performance.

At the same time, explorative learning also promotes innovation at the individual level. Employee innovation reflects the proactive search, development, and application of novel ideas in daily work [53]. Drawing on Social Learning Theory, employees acquire and apply new ideas through observation, experimentation, and interaction, which facilitates innovative behaviour. A learning-oriented environment provides the cognitive resources, skills, and psychological safety needed to encourage such behaviors [43,45]. When employees feel supported and empowered to innovate, they are more likely to propose improvements to processes, products, or services, thereby contributing to both individual and collective outcomes [54]. Empirical studies confirm that innovative employee behavior is a mechanism linking supportive learning environments with performance improvements [55,56]. Accordingly, employee innovation is expected to mediate the relationship between team explorative learning and team performance.

*H4: Team innovation mediates the relationship between team explorative learning and team performance.*

*H5: Employee innovation mediates the relationship between team explorative learning and team performance.*

#### Team explorative learning and team performance.

Explorative learning enables teams to continuously search for improvement opportunities, maintain operational flexibility, and respond effectively to environmental changes [36,37]. By fostering adaptation and complex problem-solving, explorative learning is associated with greater collective creativity and collaborative effectiveness [38]. Moreover, teams that engage in sustained exploration cultivate mutual trust and psychological safety, which facilitate open knowledge sharing, coordination, and joint decision-making [28,40]. Such environments encourage members to experiment, learn from mistakes, and refine their skills, thereby supporting the team’s ability to accomplish tasks and achieve performance objectives. While innovation processes represent important mechanisms through which learning translates into performance, explorative learning may also exert a direct influence by enhancing team capabilities, coordination, and adaptability. Taken together, these arguments suggest that team explorative learning directly contributes to team performance.

*H6: Team explorative learning has a positive effect on team performance.*

#### Customer relationship management practices as a moderator.

Team explorative learning equips members to proactively experiment, acquire new knowledge, and expand their creative capacity to address rapid market changes [35]. However, the extent to which explorative learning and innovation translate into performance may depend on the organizational context, particularly the presence of Customer Relationship Management (CRM) practices. CRM provides systematic mechanisms for gathering and interpreting customer insights, which can guide teams in aligning explorative learning with market demands [9]. Rather than directly driving performance, CRM practices shape how effectively teams utilize customer knowledge within their learning and innovation processes. Prior research suggests that CRM practices facilitate access to customer information and support knowledge integration, thereby enhancing the relevance of innovation outcomes [15,57,58]. When CRM practices are well developed, teams may be better able to incorporate customer feedback into their learning processes, thereby potentially strengthening the link between innovation and performance.

At the same time, CRM systems may also introduce structured processes and performance expectations that influence how teams engage in learning activities. In highly regulated and service-intensive contexts, such systems can emphasize consistency, standardization, and responsiveness, which may shape the balance between efficiency and experimentation. As a result, while CRM can support learning and innovation, it may also affect the extent to which explorative learning translates into performance outcomes.

CRM practices also shape the social and psychological conditions for learning and innovation. By fostering psychological safety, CRM practices can enable team members to share ideas openly, experiment, and learn from failure. CRM systems provide continuous streams of customer feedback, which may support practice-based learning and service refinement [59,60]. Leaders who leverage CRM tools may be better positioned to manage team interactions and allocate resources, ensuring that explorative learning and innovation efforts are directed toward performance-enhancing outcomes [61]. Moreover, customer-oriented leadership supported by CRM practices can reinforce customer-focused strategies, potentially strengthening the performance implications of innovation activities [62]. Even in contexts of task conflict, effective CRM practices may help teams reframe disagreements as learning opportunities rather than performance threats [63]. Taken together, these arguments suggest that CRM practices act as a boundary condition that shapes the strength of relationships among team innovation, team explorative learning, employee innovation, and team performance (Fig 1). Accordingly, we hypothesize:

**Fig 1:**
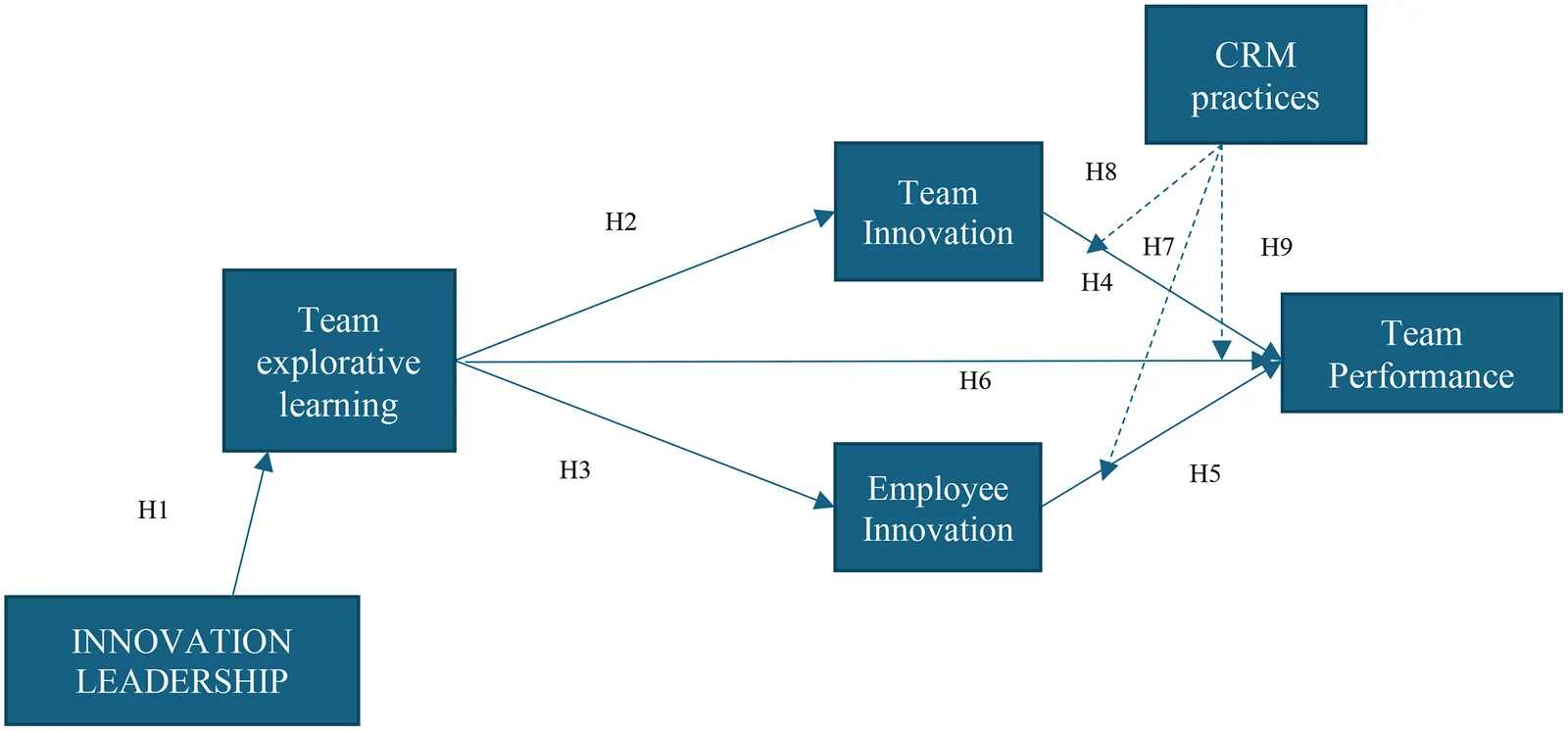
Conceptual framework. The figure illustrates the proposed relationships among the study constructs (H1to H9).

*H7: CRM practices moderate the relationship between employee innovation and team performance.*

*H8: CRM practices moderate the relationship between team innovation and team performance.*

*H9: CRM practices moderate the relationship between team explorative learning and team performance.*

## 3. Research methodology

### 3.1. Data collection

To examine the relationships among innovation leadership, team learning, team performance, and employee performance, primary data were collected through structured online surveys. The questionnaire was prepared in both English and Vietnamese to ensure clarity and inclusiveness. Following a rigorous translation procedure, the original English version was translated into Vietnamese, and both versions were administered to respondents. The sample size was determined in accordance with Hair et al. [64], who recommend that the minimum sample should be at least ten times the number of structural paths directed at a particular construct. Similarly, Hair et al. [65] argue that Partial Least Squares Structural Equation Modeling (PLS-SEM) is particularly suitable for small sample sizes and complex models, making it an appropriate choice for this study. Based on these guidelines, the minimum sample size required for the proposed model was 155. The final dataset comprised 430 valid responses, exceeding the recommended threshold.

Informed consent was obtained from all participants prior to their inclusion in the study. The consent process was informed, as all participants were provided with a written information sheet detailing the purpose of the study, research procedures, the voluntary nature of participation, confidentiality measures, and their right to withdraw at any time without penalty. Consent was obtained verbally due to the workplace-based nature of the data collection. Verbal consent was documented by the principal investigator at the time of data collection. Participation was strictly voluntary, and only individuals who explicitly agreed to participate were included in the study

Data were collected from a diverse set of organizations in Vietnam and international firms operating in the Vietnamese market, including major airlines, aviation service providers, as well as travel and hospitality firms. These organizations were selected because they employ talented leaders, foster innovation-oriented leadership, and rely on capable employees who actively contribute to strategic goals through creativity and innovation.

### 3.2. Measurement

The survey questionnaire was originally developed in English and then translated into Vietnamese following Brislin’s [66] back-translation procedure to ensure conceptual equivalence. All constructs were measured using established scales from prior studies and employed a five-point Likert scale ranging from 1 (“strongly disagree”) to 5 (“strongly agree”).

Innovation leadership was measured using five items from Lovelace et al. [67] that capture top management’s emphasis on teamwork, initiative, and clear communication. Team explorative learning constructs were assessed with five items, adapted from [35]. Team explorative learning reflects searching for and experimenting with new knowledge, whereas team exploitative learning focuses on refining and extending existing knowledge and practices.

Employee innovation was assessed using six items from [68], emphasizing individual behaviors related to idea generation, championing, and implementation. Team innovation was measured using four items from Welbourne et al. [69] that capture the extent to which teams collectively generate and implement new ideas. Team performance was measured using two items from the scale by [70]. Customer Relationship Management (CRM) practices were measured using the multi-dimensional scale developed by [71], including long-term partnership, customer involvement, joint problem-solving, and technology-enabled CRM. Control variables included respondents’ age, gender, and organizational tenure, as these demographic characteristics may influence innovation and performance outcomes.

### 3.3. Data analysis

The data analysis followed three stages. First, descriptive statistics were computed to summarize respondents’ demographic characteristics and item-level properties. Second, the measurement model was assessed for reliability and validity. Internal consistency was evaluated using Cronbach’s alpha, composite reliability (CR), and average variance extracted (AVE), with thresholds of 0.60 for loadings and CR and 0.50 for AVE [72]. Items failing to meet these criteria were excluded. Third, the structural model was tested using partial least squares structural equation modeling (PLS-SEM). Model quality was evaluated using R^2^ values, with benchmarks of 0.67 (substantial), 0.33 (moderate), and 0.19 (weak) [65]. Hypotheses were tested via bootstrapping with 5,000 resamples to estimate path coefficients, standard errors, and significance levels.

## 4. Results

### 4.1. Demographic information

Table 1 presents the demographic profile of respondents. The final sample comprised 430 valid responses. The sample comprised 51.16% males and 48.84% females. The largest age group was 31–40 years old (37.21%), followed by 41–50 years old (27.21%). Regarding work experience, 25.11% of respondents reported 2–7 years of experience, 23.02% reported 8–13 years, and the remaining respondents reported 14 years or more. In terms of education, most respondents held a university degree (70.93%). Regarding organizational position, 43.03% held Head of Department/Director or Manager positions, 42.79% were employees, 7.91% were CEOs/Founders, and 6.28% were supervisors or team leaders. Most respondents were Vietnamese (93.49%), while 6.51% were foreign respondents from 14 nationalities.

**Table 1:** Respondent demographics.

Items		Frequency	Percentage
Gender	Male	220	51.16%
	Female	210	48.84%
Age	20-30	81	18.84%
	31-40	160	37.21%
	41-50	117	27.21%
	51+	72	16.74%
Experience (year of working)	2-7	108	25.11%
	8-13	99	23.02%
	14-19	67	15.58%
	20-25	81	18.84%
	26+	75	17.44%
Education	BDA/PhD	9	2.09%
	Master	76	17.67%
	University	305	70.93%
	College	31	7.21%
	High School	9	2.09%
Position/ Title	CEO	34	7.91%
	Head of Department/Director	100	23.26%
	Manager	85	19.77%
	Supervisor/ leader	27	6.28%
	Employee	184	42.79%
Nationality 15 nationalities	Vietnamese	402	93.49%
	Foreigner (14 nationalities)	28	6.51%

### 4.2. Measurement model assessment

Table 2 reports the reliability and convergent validity of the study constructs. The constructs include Innovation Leadership (IL), Team Explorative Learning (TEL), Team Innovation (TI), Employee Innovation (EI), Team Performance (TP), and Customer Relationship Management (CRM) practices. The CRM practices dimensions include Customer Involvement (CI), Joint Problem-Solving (JPS), Long-Term Partnership (LTP), and Technology-Based CRM(TB). Reliability and convergent validity were assessed using outer loadings, Cronbach’s alpha, composite reliability (CR), and average variance extracted (AVE). Outer loadings above 0.70 indicate satisfactory indicator reliability. Cronbach’s alpha and CR values above 0.70 indicate acceptable internal consistency reliability, while AVE values above 0.50 indicate acceptable convergent validity.

**Table 2:** Reliability and convergent validity results of constructs.

Factors	Indicators	Outer loadings	Cronbach’s Alpha	CR	AVE
Innovation Leadership (IL)	IL1	0.810	0.904	0.905	0.723
	IL2	0.865			
	IL3	0.862			
	IL4	0.857			
	IL5	0.857			
Team Explorative Learning (TEL)	TEL1	0.867	0.931	0.932	0.784
	TEL2	0.896			
	TEL3	0.901			
	TEL4	0.874			
	TEL5	0.887			
Team Innovation (TI)	TI1	0.910	0.935	0.936	0.837
	TI2	0.913			
	TI3	0.926			
	TI4	0.910			
Employee Innovation (EI)	EI1	0.867	0.922	0.922	0.719
	EI2	0.893			
	EI3	0.805			
	EI4	0.819			
	EI5	0.855			
	EI6	0.847			
Team Performance (TP)	TP1	0.962	0.915	0.916	0.921
	TP2	0.958			
CRM Practices	CRM- CI	0.849	0.896	0.914	0.761
	CRM- JPS	0.878			
	CRM-LTP	0.891			
	CRM-TB	0.870			

As shown in Table 2, all indicator outer loadings exceeded the 0.70 threshold, demonstrating satisfactory indicator reliability. Cronbach’s alpha values ranged from 0.896 to 0.935, and composite reliability (CR) values ranged from 0.905 to 0.936, both well above the recommended cutoff of 0.70 [72], confirming strong internal consistency. In addition, average variance extracted (AVE) values ranged from 0.719 to 0.921, exceeding the 0.50 benchmark and thereby establishing convergent validity. Team performance (TP) reported the highest AVE (0.921) among the constructs, indicating that its items captured a substantial proportion of shared variance. Overall, these results confirm that all constructs demonstrated satisfactory reliability and convergent validity, supporting the adequacy of the measurement model.

Table 3 reports the Heterotrait-Monotrait Ratio (HTMT) values to assess discriminant validity among the constructs. The constructs include Customer Relationship Management (CRM), Employee Innovation (EI), Innovation Leadership (IL), Team Explorative Learning (TEL), Team Innovation (TI), and Team Performance (TP). The interaction terms CRM × EI, CRM × TI, and CRM × TEL represent the moderating effects of CRM practices on the relationships between employee innovation and team performance, team innovation and team performance, and team explorative learning and team performance, respectively. HTMT values below 0.90 indicate satisfactory discriminant validity [73].

**Table 3:** HTMT results.

	CRM	EI	IL	TEL	TI	TP	CRM x EI	CRM x TI
CRM								
EI	0.617							
IL	0.675	0.529						
TEL	0.656	0.697	0.716					
TI	0.655	0.811	0.637	0.875				
TP	0.598	0.641	0.570	0.716	0.709			
CRM x EI	0.157	0.156	0.087	0.157	0.133	0.109		
CRM x TI	0.184	0.134	0.165	0.236	0.222	0.120	0.814	
CRM x TEL	0.078	0.158	0.138	0.244	0.238	0.190	0.709	0.863

In this study, all HTMT values are below the conservative threshold of 0.90, ranging from 0.078 to 0.875. The highest value (0.875) was observed between Team Explorative Learning (TEL) and Team Innovation (TI), which is still within acceptable limits. Additionally, interaction terms such as CRM × EI, CRM × TI, and CRM × TEL also show low HTMT values, further supporting discriminant validity. These results confirm that all constructs in the model are empirically distinct from one another, indicating that discriminant validity is satisfactorily established.

### 4.3. Structural model assessment

Table 4 presents the results of the structural model analysis, including path coefficients, t-statistics, p-values, and R^2^ values for the dependent variables. Path coefficients indicate the direction and strength of the relationships, while R^2^ values indicate the explained variance of the endogenous constructs. The interaction terms CRM × EI, CRM × TI, and CRM × TEL represent the moderating effects of CRM practices. The results confirm the statistical significance of most hypothesized relationships.

**Table 4:** Hypothesis testing results.

		Beta – Original sample (O)	T statistics (|O/STDEV|)	P values	R2
H1	IL - > TEL	0.657	14.528	0.000	0.430
H2	TEL - > TI	0.818	38.387	0.000	0.669
H3	TEL - > EI	0.647	18.164	0.000	0.418
H4	TEL - > TI - > TP	0.153	2.330	0.020	
H5	TEL - > EI - > TP	0.080	2.225	0.026	
H6	TEL - > TP	0.529	10.105	0.000	0.430
H7	CRM x EI - > TP	−0.047	1.000	0.317	
H8	CRM x TI - > TP	0.273	3.605	0.000	
H9	CRM x TEL - > TP	−0.229	3.780	0.000	

Specifically, H1 (IL → TEL) is supported, with a substantial and significant path coefficient (β = 0.657, p = 0.000), indicating that innovation leadership positively influences team explorative learning (R^2^ = 0.430). H2 (TEL → TI) shows a very strong and significant effect (β = 0.818, p = 0.000), with a high explanatory power for team innovation (R^2^ = 0.669). Similarly, H3 (TEL → EI) is significant (β = 0.647, p = 0.000), contributing to employee innovation (R^2^ = 0.418). Mediation paths H4 (TEL → TI → TP) and H5 (TEL → EI → TP) are statistically significant (p < 0.05), confirming partial mediation effects. H6 (TEL → TP) is also significant (β = 0.529, p = 0.000), indicating a direct effect of team explorative learning on team performance (R^2^ = 0.430).

Among the moderating effects, H7 (CRM × EI → TP) is not significant (p = 0.317), suggesting that CRM practices do not significantly moderate the relationship between employee innovation and team performance. However, H8 (CRM × TI → TP) is significant (β = 0.273, p = 0.000), indicating that CRM practices strengthen the effect of team innovation on team performance. Interestingly, H9 (CRM × TEL → TP) is significant but negative (β = –0.229, p = 0.000), suggesting that CRM practices may weaken the direct effect of team explorative learning on team performance. These findings highlight the central role of explorative learning in driving innovation and performance and the nuanced role of CRM practices as moderators (Fig 2).

**Fig 2:**
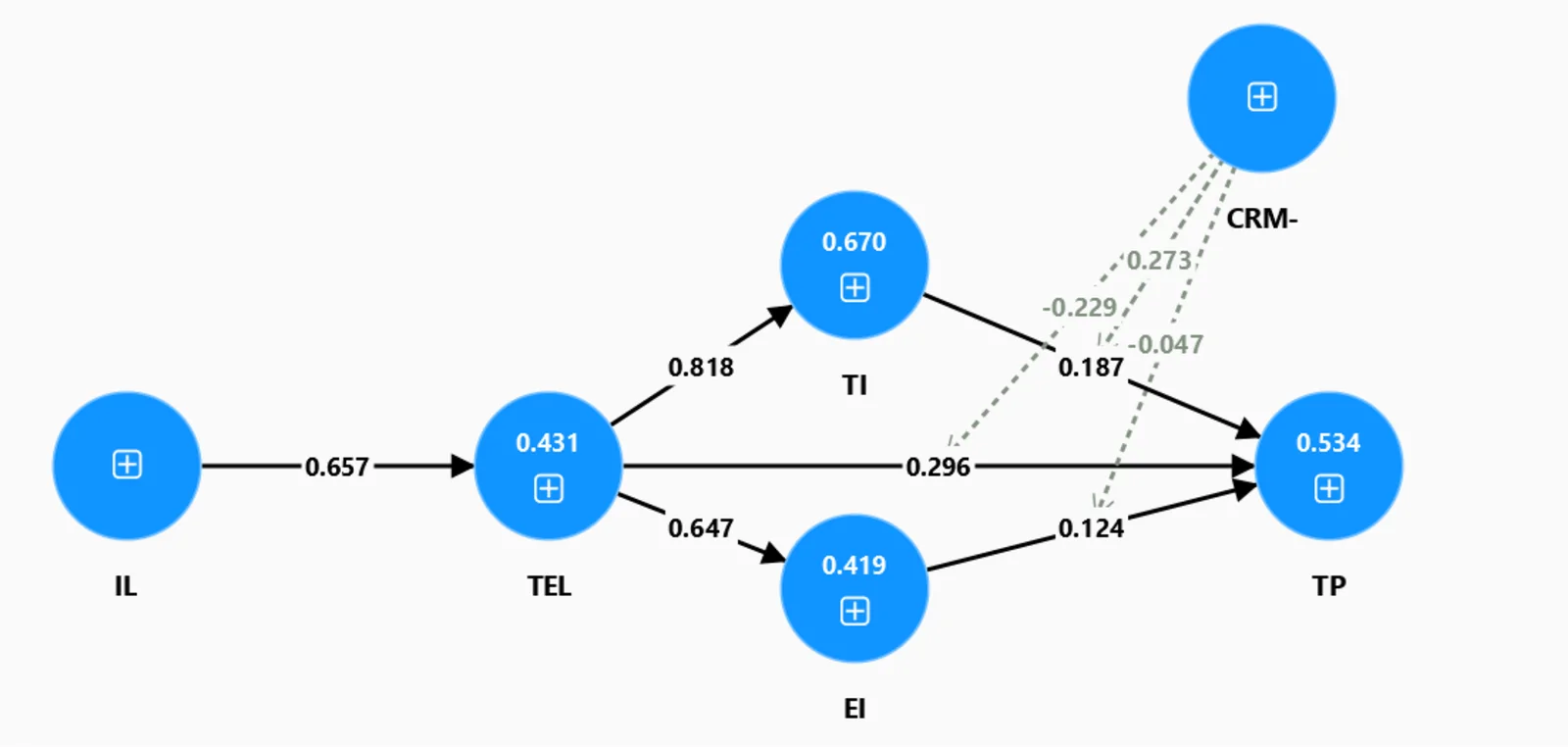
The PLS_SEM model. The figure presents the estimated path coefficients and values of the structural model.

## 5. Discussion and contributions

### 5.1. Discussion

This study examined how innovation leadership and team explorative learning shape innovation outcomes and team performance in Vietnam’s aviation and tourism sector. Overall, the empirical results provide robust support for the hypothesized model, with the exception of H7. In particular, team explorative learning emerged as a key mechanism through which innovation leadership drives team performance, while CRM moderated the link between team innovation and performance. Consistent with prior research, innovation leadership significantly enhanced team explorative learning (H1: β = 0.657, p = 0.000), echoing Bucic et al. [74], Kremer et al. [31], and Zaccaro et al. [34]. These findings highlight that visionary and supportive leaders create psychological safety and intellectual stimulation, which, in turn, encourage knowledge sharing and experimentation.

Team explorative learning was found to strongly predict both team innovation (H2: β = 0.818, p = 0.000) and employee innovation (β = 0.647, p = 0.000), supporting the arguments of van den Hoed et al. [39] and Fang et al. [38] that continuous exploration is central to unlocking creativity at both collective and individual levels. Team explorative learning also exerted direct effects on team performance (H6: β = 0.529, p = 0.000) and indirect effects via team innovation (H4: β = 0.153, p = 0.020) and employee innovation (H5: β = 0.080, p = 0.026). These results resonate with the multilevel innovation frameworks proposed by West and Farr [47] and Anderson et al. [49], reinforcing the notion that learning must be translated into concrete innovations to deliver performance improvements. Importantly, these findings suggest that explorative learning generates new knowledge and provides the foundation for sustaining competitive advantage in dynamic service environments, where rapid adaptation is essential for survival.

The moderating effects of CRM produced mixed results. Contrary to expectations, CRM did not significantly moderate the relationship between employee innovation and performance (H7, p = 0.317), diverging from Battor and Battor [15], who emphasized the role of customer-focused innovation. A plausible explanation lies in aviation, where safety regulations and standardized operating procedures often constrain employee creativity. This highlights a critical tension between compliance-driven environments and the need for individual innovation, suggesting that fostering creativity in such industries requires structural and cultural shifts beyond CRM practices alone. By contrast, CRM significantly strengthened the relationship between team innovation and performance (H8, β = 0.273, p = 0.000), suggesting that structured customer feedback systems can amplify the effectiveness of collective innovation, in line with Donate and Guadamillas [57]. This underscores the value of CRM not merely as a marketing tool but as a strategic enabler of team-based innovation. CRM negatively moderated the direct link between team explorative learning and performance (H9, β = –0.229, p = 0.000). This finding can be understood through the tension between customer alignment and explorative flexibility in highly regulated service contexts. This counterintuitive finding implies that excessive reliance on CRM structures may rigidify learning processes by overly aligning them with customer expectations. In such contexts, CRM may introduce structural and cognitive constraints that shift team behavior toward efficiency and predictability, thereby potentially reducing the effectiveness of explorative learning. As Xiang et al. [60] caution, overemphasizing external feedback without balancing autonomy can suppress adaptive learning behaviors. This critical insight highlights the need for managers to carefully calibrate CRM, leveraging customer insights while preserving flexibility for creative experimentation.

### 5.2. Theoretical contributions

This study makes several contributions. First, this study advances Social Exchange Theory (SET) and Social Learning Theory (SLT) by explicating the underlying mechanism through which innovation leadership translates into innovation outcomes. While prior research has established that leadership fosters innovation [31], the process through which this influence unfolds remains insufficiently understood. Drawing on SET and SLT, this study identifies team explorative learning as a key mediating mechanism that converts leadership-driven social exchange relationships and observational learning processes into innovative outcomes. Importantly, the study extends existing research by demonstrating that this mechanism operates across multiple levels, linking innovation leadership to both team-level and employee-level innovation. In doing so, it provides a more fine-grained understanding of how leadership-driven learning processes generate innovation within teams.

Second, this study advances the team learning literature by conceptualizing and empirically demonstrating team explorative learning as a dynamic capability that drives innovation and performance. Rather than viewing learning as a static or isolated activity, the findings show that explorative learning functions as an enabling mechanism through which teams generate both team-level and employee-level innovation outcomes. The identification of dual mediation pathways via team innovation and employee innovation offers a more nuanced understanding of how learning translates into performance, highlighting that innovation emerges through both collective and individual-level processes. Importantly, by integrating individual- and team-level innovation within a single empirical model, this study provides a multilevel perspective on learning-based value creation, demonstrating how explorative learning simultaneously shapes outcomes across levels of analysis. In doing so, it moves beyond prior research that has typically examined these processes in isolation and offers a more comprehensive account of how team learning capabilities unfold in complex organizational settings.

Third, this study advances research on Customer Relationship Management (CRM) by positioning it as a critical boundary condition in the learning- innovation- performance relationship. Moving beyond the dominant view of CRM as uniformly beneficial (e.g., [15,57,60]), the findings reveal a more complex and paradoxical role. Specifically, CRM strengthens the innovation pathway, enhancing the effects of team innovation on performance while weakening the direct impact of team explorative learning on performance. This dual effect highlights a fundamental tension between customer alignment and explorative flexibility: while CRM facilitates translating formalized innovation into performance, it may constrain the immediate value of explorative learning by emphasizing standardization and responsiveness to customer expectations. By uncovering these opposing effects, the study reconceptualizes CRM practices not simply as a supportive tool, but as a contingent organizational mechanism whose value depends on how it is embedded within the innovation process. This perspective offers a more nuanced understanding of CRM practices, demonstrating that its impact varies across different stages and forms of innovation-related activity.

Fourth, the study contributes contextually by situating these mechanisms within Vietnam’s aviation and tourism industries, which are characterized by volatility, regulatory pressures, and post-pandemic recovery. This setting provides a critical test of established theories, illustrating how innovation leadership, team learning, and CRM practices operate in an emerging-market service environment distinct from those commonly studied in the technology and education sectors [11,12]. In doing so, the research extends innovation and learning theories to global service contexts where safety, customer orientation, and adaptability must be balanced. Accordingly, the study contributes by reinforcing, extending, and contextualizing existing theory within a setting that presents distinctive operational and managerial conditions, rather than by advancing theoretical novelty for its own sake.

### 5.3. Managerial implications

Beyond its theoretical contributions, this study offers several important implications for practitioners in service-intensive, customer-facing industries, particularly in high-risk, highly regulated contexts such as aviation and tourism. First, organizations should prioritize developing innovation leadership capabilities. Leaders who encourage experimentation, tolerate failure, and foster psychological safety can substantially strengthen team learning and innovation. Training and leadership development programs should therefore emphasize competencies in open communication, coaching, and support for innovative behaviors.

Second, managers are encouraged to design teams that maximize explorative learning. Providing autonomy, access to diverse sources of information, and opportunities for reflection can foster curiosity and continuous learning, thereby enhancing creativity and performance. Importantly, such autonomy should be embedded within clearly defined procedural boundaries to ensure that learning activities enhance, rather than disrupt, operational reliability. Embedding these practices into daily routines will help teams respond more effectively to complex and dynamic challenges.

Third, while CRM systems can enhance innovation effectiveness particularly at the team level their design and implementation must remain flexible. Overly rigid CRM structures may inadvertently constrain learning and limit the benefits of explorative behaviors. Organizations should therefore adopt CRM practices that integrate customer insights iteratively and adaptively, ensuring that feedback informs innovation without restricting team autonomy.

Finally, this study highlights the importance of viewing innovation as a multi-level phenomenon that requires alignment across leadership behaviors, team learning processes, and system-level structures such as CRM. Such alignment is particularly critical in regulated service environments, where organizations must simultaneously ensure compliance, consistency, and service differentiation. Achieving such strategic alignment can unlock the full potential of innovation to drive sustainable performance in highly dynamic service environments.

## 6. Conclusion, limitations, and future research

This study advances our understanding of the mechanisms through which innovation leadership enhances team performance in service-intensive contexts, particularly by promoting team explorative learning and stimulating both individual and collective innovation. Drawing on Social Exchange Theory and Social Learning Theory, the results show that innovation leadership cultivates a learning environment that encourages experimentation and knowledge sharing, thereby driving innovation outcomes that improve overall team performance. The findings also highlight the dual role of CRM practices as a boundary condition: while it strengthens the link between team innovation and performance, it unexpectedly attenuates the direct effect of team learning on performance, suggesting potential tensions between structured customer orientation and autonomous team learning.

Several limitations should be noted. First, the cross-sectional design limits causal inference, and longitudinal studies are needed to better capture the temporal dynamics of learning and innovation. Second, this study relies exclusively on self-reported survey data collected from the same respondents for both predictor and outcome variables. Although statistical techniques were applied to assess and control for common method variance, the possibility of single-source bias, self-enhancement, and social desirability effects cannot be fully ruled out. This issue may have contributed to the relatively high path coefficients observed in the model, and the findings should therefore be interpreted with appropriate caution. Future research is encouraged to incorporate multi-source designs, such as combining employee-rated predictors with supervisor-rated innovation and performance outcomes, as well as objective performance indicators, to strengthen the robustness of the results. Third, the sample was drawn exclusively from the Vietnamese aviation and tourism sector, which may limit generalizability to other industries or cultural settings. In addition, the sample includes respondents from different organizational types, including airlines, ground services, catering, travel agencies, and hotels. Although these organizations operate within the broader aviation and tourism ecosystem, they may differ in terms of organizational structure, regulatory conditions, customer interface, and innovation dynamics. Because the present study analyzed them as a combined sample, potential sectoral and organizational differences were not explicitly examined. Therefore, the findings should be generalized with caution. Future research may benefit from comparing different organizational types, regulatory contexts, and domestic versus international firms to provide richer contextual insights and stronger external validity. Future studies could examine additional contextual moderators, such as organizational culture, team autonomy, or technological capability. Exploring how different CRM configurations, flexible versus rigid, interact with team learning processes may provide further insights. Cross-country and cross-sector investigations would enhance external validity and offer guidance for global innovation strategies.
